# Supplementary material for: Experimental evolution of hybrid populations to identify Dobzhansky–Muller incompatibility loci
Source: Ecol Evol. 2024 Feb 8;14(2):e10972. doi: 10.1002/ece3.10972 (PMC10851027; doi:10.1002/ece3.10972)
Supplement: Supplementary file 1 — Appendix S1 [file ECE3-14-e10972-s001.docx]

# Supplementary

**
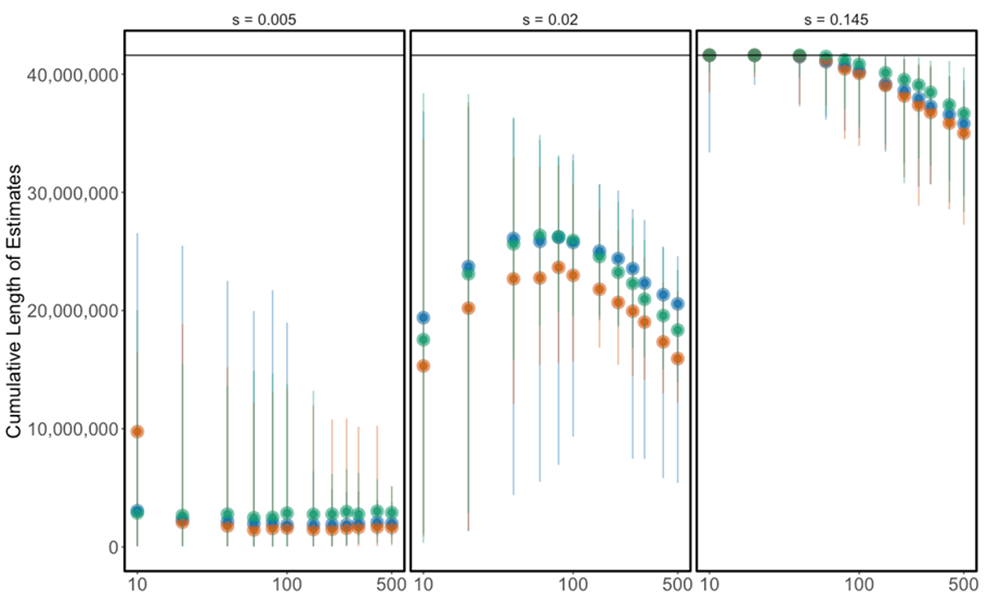
**

**
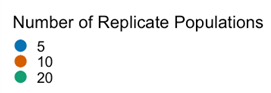
**

**Figure S1.** Cumulative length of mapped DMI estimates as a function of the duration of experimental evolution (log scale generations) for 20 DMI loci that express one-way pairwise incompatibilities, inferred from 5, 10, and 20 replicate populations. Error bars correspond to 2.5^th^ to 97.5^th^ quantiles.


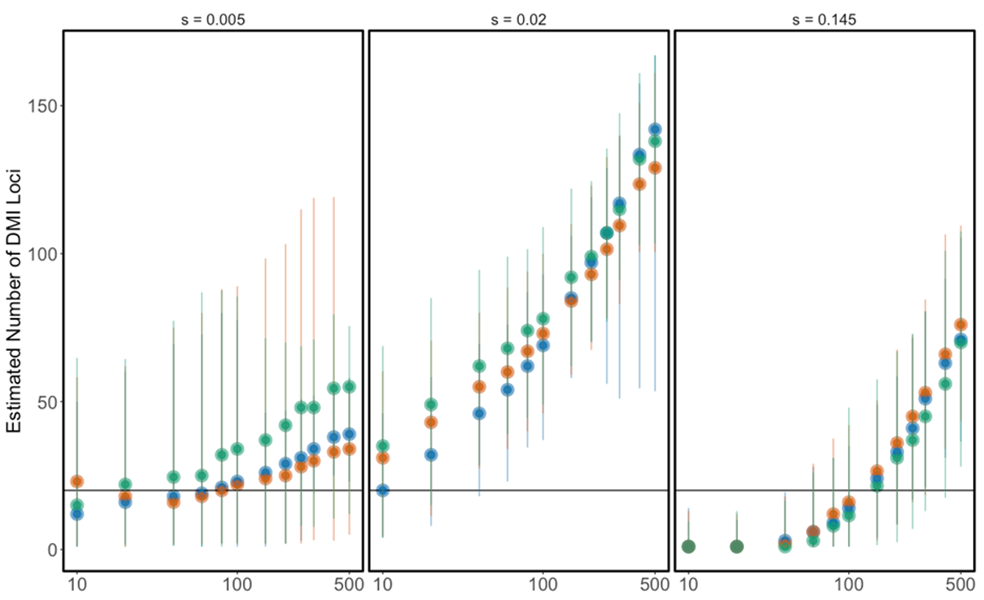


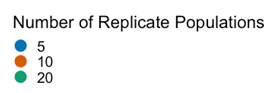


**Figure S2.** Estimated number of DMI loci as a function of the duration of experimental evolution (log scale generations) for 20 DMI loci that express pairwise one-way incompatibilities, inferred from 5, 10, and 20 replicate populations. Error bars correspond to 2.5^th^ to 97.5^th^ quantiles.


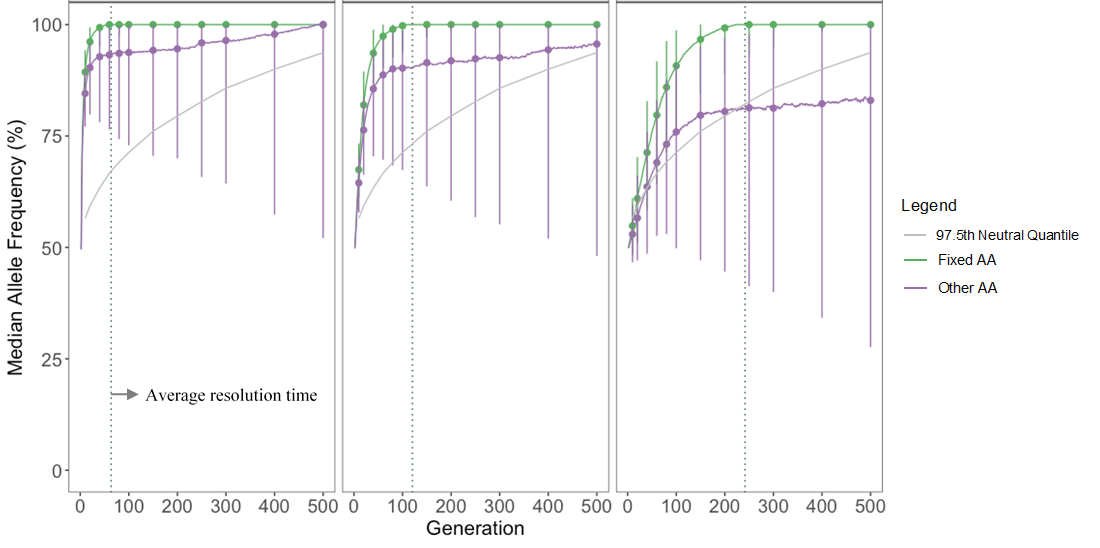


**Figure S3.** Median allele frequencies at each locus in a DMI pair for one-way incompatibilities. The ancestral allele (AA) that fixed first in each population (green line) causes DMI resolution. The frequency of the other ancestral allele at the other locus (purple line) are then subject only to genetic drift in subsequent generations. Frequency data were collected at each generation 1 to 500, collected separately from the simulation runs used to generate estimates, but resembled them in every way except for the exclusion of the 20,000 neutral loci. In each population, the fixed ancestral allele can either be from parental species 1 or 2. Median values are calculated from 1000 replicate populations that contain a one-way dominant DMI with a selection coefficient of 0.05, 0.2, or 0.8. The error bars for 12 timepoints are the frequency 95% interquantiles ranging from the 2.5^th^ and 97.5^th^ quantiles. The dashed vertical line corresponds to the average timepoint at which DMI resolution occurs across replicates. The gray curve indicates the 97.5^th^ quantiles of allele frequencies from 1000 populations lacking fitness-affecting DMI loci.
